# Interspecies extrapolation by physiologically based pharmacokinetic modeling

**DOI:** 10.17179/excli2015-759

**Published:** 2015-12-21

**Authors:** Ahmed Ghallab

**Affiliations:** 1 Forensic Medicine and Toxicology Department, Faculty of Veterinary Medicine, South Valley University, Qena, Egypt

## ⁯⁯⁯

Recently, Christoph Thiel and colleagues from Aachen University published an improved physiologically based pharmacokinetik modeling (PBPK) technique for mouse to human extrapolation (Thiel et al., 2015[18]). This publication will be awarded by the Ebert Prize 2016 of the American Pharmacist Association, which represents the oldest pharmacy award in the United States.

The translation of preclinical knowledge often generated in mice to first-in-human studies represents a critical step (Thiel et al., 2015[18]). More than 30 % of developmental compounds fail due to interspecies differences. To improve the situation, the authors used PBPK modeling to predict human plasma concentration-time profiles based on mouse data. The study was based on 10 exemplary drugs for which a comprehensive pharmacokinetic database is available. Mouse to human extrapolation was achieved by adjustment of four model parameter domains (Thiel et al., 2015[18]):

1. species-specific physiology, such as differences in organ size, perfusion, etc.
2. the species-specific non-protein bound fraction of the test compound,
3. kinetic parameters, such as V_max_ and K_M_ for the primary route of excretion, and
4. tissue-specific gene expression of the metabolizing key enzymes and transporters.

The authors start with a naïve extrapolation where humans are considered as 'large mice' where the same dose per body weight was administered (Thiel et al., 2015[18]). This naïve extrapolation usually resulted in predictions that strongly deviate from the real human situation. Next the authors showed that knowledge-based adjustment of each of the four model domains leads to an improvement and allows predictions which closely resemble the measured situation in humans. A limitation of the current approach is that gene expression data were used to adjust for interspecies differences in metabolism. In future, predictions may become even more accurate if RNA based data could be replaced by metabolic activities. 

Interspecies differences represent a major problem in toxicology (Dohnal et al., 2014[5]; Bernauer et al., 2000[1]; Brüning et al., 2014[2]; Gerbracht and Spielmann, 1998[9]; Unkila et al., 1995[19]; Leist and Hartung, 2013[15]). Rodent to human comparisons have often been performed by comparing data in human and mouse or rat hepatocytes (Carmo et al., 2004[3], 2005[4]; Reder-Hilz et al., 2004[16]; Hewitt et al., 2007[12]; Gebhardt et al., 2003[8]; Godoy et al., 2013[10]; Hengstler et al., 1999[11]). However, differences in metabolism represent only one of several aspects which can explain interspecies differences. 

PBPK modeling has been used since long to predict absorption, distribution, metabolism and excretion (Sterner et al., 2013[17]; Lee et al., 2007[14]; Jonsson et al., 2001[13]; El-Masri et al., 1996[7][6]). However, the approach presented by Thiel and colleagues (2015[18]), in which all parameter domains relevant for interspecies differences can be stepwise adjusted, represents an important step to improve extrapolations from rodent models to predict the human situation.
